# Dipeptidyl Peptidase 4: A New Link between Diabetes Mellitus and Atherosclerosis?

**DOI:** 10.1155/2015/816164

**Published:** 2015-06-04

**Authors:** Wellington Santana da Silva Júnior, Amélio Fernando de Godoy-Matos, Luiz Guilherme Kraemer-Aguiar

**Affiliations:** ^1^Postgraduate Program in Clinical and Experimental Physiopathology (FISCLINEX), State University of Rio de Janeiro, 20551-030 Rio de Janeiro, RJ, Brazil; ^2^Diabetes Department, State Institute of Diabetes and Endocrinology (IEDE), 21330-683 Rio de Janeiro, RJ, Brazil; ^3^Metabolism Department, IEDE, Catholic University, 21330-683 Rio de Janeiro, RJ, Brazil; ^4^Obesity Unit, Division of Endocrinology, Department of Internal Medicine, Faculty of Medical Sciences, Policlínica Piquet Carneiro (UERJ), 20551-030 Rio de Janeiro, RJ, Brazil; ^5^Laboratory for Clinical and Experimental Research on Vascular Biology, Biomedical Center, State University of Rio de Janeiro, 20550-013 Rio de Janeiro, RJ, Brazil

## Abstract

Type 2 diabetes mellitus (T2DM) has become one of the most prevalent noncommunicable diseases in the past years. It is undoubtedly associated with atherosclerosis and increased risk for cardiovascular diseases. Incretins, which are intestinal peptides secreted during digestion, are able to increase insulin secretion and its impaired function and/or secretion is involved in the pathophysiology of T2DM. Dipeptidyl peptidase 4 (DPP4) is an ubiquitous enzyme that regulates incretins and consequently is related to the pathophysiology of T2DM. DPP4 is mainly secreted by endothelial cells and acts as a regulatory protease for cytokines, chemokines, and neuropeptides involved in inflammation, immunity, and vascular function. In T2DM, the activity of DPP4 seems to be increased and there are a growing number of *in vitro* and *in vivo* studies suggesting that this enzyme could be a new link between T2DM and atherosclerosis. Gliptins are a new class of pharmaceutical agents that acts by inhibiting DPP4. Thus, it is expected that gliptin represents a new pharmacological approach not only for reducing glycemic levels in T2DM, but also for the prevention and treatment of atherosclerotic cardiovascular disease in diabetic subjects. We aimed to review the evidences that reinforce the associations between DPP4, atherosclerosis, and T2DM.

## 1. Introduction

Atherosclerosis is the leading cause of death and an important cause of morbidity in patients with type 2 diabetes mellitus (T2DM) [1]. However, the mechanisms responsible for the accelerated atherosclerosis observed in T2DM are not yet fully understood [2]. Reduction in the bioavailability of nitric oxide (NO) in the periendothelial environment, which characterizes endothelial dysfunction, is the earliest event in the development of atherosclerosis [2]. Since the occurrence of endothelial dysfunction may be observed before the development of T2DM, it is suggested that these two entities, T2DM and atherosclerosis, may have common pathogenic mechanisms which enhances the possibility of a causal relationship between them [3]. Not only reduced endothelial NO bioavailability, but also inflammation has a role in the promotion of vascular damage in T2DM and has been receiving special attention [4]. Some recent findings add knowledge in these intricate mechanisms and relate the enzyme dipeptidyl peptidase 4 (DPP4) with them.

T2DM has a complex pathophysiology, mainly characterized by insulin resistance (IR) in fat, muscle, and liver tissues associated with pancreatic *α* and *β* cell dysfunctions [5, 6]. However, other factors play a role in the development of T2DM. Among them, stands out the incretin deficiency/resistance [5]. Glucose-dependent insulinotropic polypeptide (GIP) and glucagon-like peptide 1 (GLP-1) are the main incretins secreted by the gastrointestinal tract soon after a meal ingestion [7]. Both are able to enhance insulin secretion in a glucose-dependent fashion while suppressing glucagon secretion [6], although GIP has a more complex relationship with glucagon. Actually, GIP acts as a hormone that stabilizes glucose in T2DM by increasing glucagon response during hypoglycemia, the secretion rate of insulin during hyperglycemia, and both mechanisms when fasting glucose levels are around 8 mmol/L [8].

The state of incretin deficiency/resistance reflects the impairment of the “incretin effect,” defined as the amplification of insulin secretion in response to an oral glucose load when compared to the insulin response observed after the same glycemic levels achieved after intravenous glucose infusion [9]. Both GIP and GLP-1 have short half-lives, since they are rapidly degraded by DPP4, an ubiquitous enzyme found in soluble form in plasma or as a membrane component of many cells [10], including endothelial cells [11]. The findings of increased concentrations and activity of DPP4 in patients with diabetes [12–15] may justify, at least partially, the status of incretin deficiency/resistance related to T2DM.

In recent years, new drugs for the treatment of T2DM have emerged into the market, among which the gliptins stand out. These drugs act through the inhibition of DPP4; consequently they are able to ameliorate the incretin deficiency and to attenuate the hyperglucagonemia, two important aspects in the pathophysiology of the T2DM [6]. Gliptins and the GLP-1 receptors agonists comprise the group of incretin-based therapies for T2DM [7].

An important point to emphasize is the capacity of DPP4 to inactivate not only incretins, but also a number of cytokines, chemokines, and neuropeptides involved in inflammation, immunity, and vascular function [16]. Furthermore, the pharmacological inhibition of DPP4 is associated with attenuation of endothelial dysfunction and atherogenesis [17] and also with reduction of inflammatory markers [18]. Considering the higher concentrations and activity of DPP4 in patients with diabetes when compared to nondiabetic subjects [12–15], it is possible that DPP4 constitutes a new link between diabetes and atherosclerosis.

## 2. DPP4: A Regulatory Serine Exopeptidase

DPP4, also known as adenosine deaminase binding protein or cluster of differentiation 26 (CD26), is a serine exopeptidase able to inactivate various oligopeptides through the removal of N-terminal dipeptides. Its chemical structure remained relatively preserved over evolutionary process and it has been observed in very distinct species, including prokaryotes and eukaryotes organisms [19].

In humans, the DPP4 gene is located on chromosome 2q23, encoding a protein of 766 amino acids [19]. Immediately after its synthesis, DPP4 is incorporated into the plasma membrane of many cell types. It is a type II surface protein, which means that the greatest part of its structure, including the C-terminal domain, is in the extracellular portion [20]. However, under certain stimuli, like IR, tumor necrosis factor *α* (TNF-*α*), and chronic low-grade inflammation, DPP4 can be released from the membrane, constituting a soluble form [15, 20, 21].

DPP4 is widely expressed in many specialized cell types and has distinct functions independently of its form, anchored to the membrane or in soluble form. As a cell surface protein, it acts as a regulatory protease and participates in complex mechanisms such as cell-cell interaction and activation of transduction pathways of intracellular signals. The soluble form of DPP4 appears to be derived primarily from endothelial cells, epithelial cells, and leukocytes and, as previously mentioned, it is also endowed with enzymatic activity [20, 28].

DPP4 activation involves a dimerisation process with the formation of a homodimer. The activity of its monomeric form is not significant [29] which possibly explains the apparent dissociation observed between serum levels of DPP4 and its enzymatic activity in humans.

Like other serine proteases, this enzyme has no absolute specificity, although it has a better affinity for oligopeptides composed of proline, hydroxyproline, or alanine as the penultimate residue [19]. DPP4 has currently many known substrates (see Table 1).

Since DPP4 has a wide capacity to act in various peptides, it appears to regulate several physiological pathways involved not only in glucose homeostasis but also in inflammation, immunity, and vascular and cardiac functions [16, 30]. These properties reinforce the hypothesis that this enzyme may act on regulatory mechanisms of endothelial function and inflammatory processes by incretin-dependent and also incretin-independent pathways.

## 3. The Role of DPP4 in Diabetes Pathophysiology and Related Complications

Since incretin hormones are rapidly degraded by DPP4, it is reasonable to assume that an increase in DPP4 level and/or enzymatic activity may contribute to the impaired incretin effect observed in patients with T2DM [12]. Preliminary studies assessing DPP4 activity in patients with T2DM have shown contradictory results such as reduced [31, 32] or increased activity [12, 13, 30]. However, these disparate results may have occurred due to the use of drugs such as metformin and glitazones, which are both able to promote a decrease in DPP4 activity [33–35].

A more recent study [15] that compared serum levels and plasma activity of the DPP4 among patients with T2DM and healthy subjects showed significant higher levels and activity of the DPP4 in those with diabetes than in controls, but only after excluding patients treated with metformin and/or glitazones.

Interestingly, not only patients with T2DM but also those at prediabetes status appear to have an impairment of incretin effect. It has been shown that patients with prediabetes exhibit decreased GLP-1 and unaltered GIP levels, as compared to those with normal glucose tolerance [36]. Therefore, it is suggested that the reduction in GLP-1 levels and/or a greater GIP resistance may contribute to impairment in insulin secretion in patients with prediabetes [36, 37].

Regardless of the glucose tolerance status (normal glucose tolerance, prediabetes, or T2DM), a 4-year longitudinal study showed that baseline DPP4 activity and GLP-1 were negatively associated. Moreover, DPP4 activity was an independent predictor of risk for developing prediabetes (relative risk (RR): 2.77; 95% confidence interval (CI): 1.38–5.55; *P* < 0.01) and T2DM (RR: 5.10; 95% CI: 1.48–17.61; *P* < 0.05) after adjustment for confounding risk factors [38]. The hypothesis that the changes in incretins in prediabetes are directly related to DPP4 seems to be a plausible one. Considering the cardiovascular (CV) complications of diabetes, this hypothesis acquired even greater relevance since a number of studies provided evidence for the pleiotropic effects of GLP-1 on the CV system [39–43].

Advanced glycation end products (AGEs) are a well-known consequence of the chronic hyperglycemia related to uncontrolled diabetes. They are formed by nonenzymatic reaction between reducing sugars and amino groups of proteins, lipids, and nucleic acids. The interaction between AGE and its receptor (RAGE) elicits oxidative stress generation, thereby evoking proliferative, inflammatory, and fibrotic reactions, which impairs structural integrity and function of many proteins. An active participation of AGEs-RAGE axis in the accelerated atherosclerosis observed in diabetes was already denoted [44]. In respect of DPP4, it was demonstrated that levels of AGEs are independently correlated with the levels of this enzyme [45]. Curiously, AGEs enhance the expression of DPP4 and its release [45], while DPP4 increases RAGE gene expression [46], suggesting the existence of a cross talk between the AGEs-RAGE axis and DPP4 in the pathogenesis of diabetes-associated complications [44].

## 4. Interaction between DPP4 and Endothelium

Endothelial cells independent of their site, that is, microvascular or macrovascular compartments, are probably the main endogenous source of DPP4. Its activity at endothelial milieu appears to be more substantial than that of the circulating form [47]. Endothelial cells from microvascular compartment showed significant increased expression of DPP4, as well as enzymatic activity, after chronic exposure to high glucose concentrations* in vitro* [48]. Microcirculation is the site of tissue nutrition, of gas exchange, and also of removal of cellular excreta and, although DPP4 is present in all vascular beds, hyperglycemia is able to increase the DPP4 activity only from the endothelial cells at the microvascular compartment [49].

*In vivo* studies added important knowledge about the action of gliptins on atherosclerosis and, interestingly, have suggested that DPP4 inhibition has GLP-1-independent effects, possibly through regulation of other enzyme substrates, acting on attenuation of endothelial dysfunction and atherogenesis [17]. Among them, the chemokine stromal cell-derived factor 1*α* (SDF-1*α*) has received special attention. SDF-1*α* is highly expressed by the human bone marrow endothelium and it is implicated in the migration, proliferation, differentiation, and survival of many cell types, including human hematopoietic stem cells and progenitor cells [50, 51]. This chemokine has its own receptor, named CXCR4, which is a seven-transmembrane G-protein receptor widely expressed by a variety of cell types, including hematopoietic, endothelial, and stromal cells [51]. The SDF-1*α*-CXCR4 axis participates in the recruitment of endothelial progenitor cells (EPCs) from bone marrow to areas of vascular damage, constituting an important mechanism of vascular repair [52, 53]. There is a positive relationship between the number of EPCs and the improvement in vascular repair and, actually, EPCs are used as a marker to assess endothelial function. It was demonstrated that DPP4 inhibition with a gliptin (sitagliptin) increased the number of EPCs, possibly due to a concomitant increase on the levels of SDF-1*α* [52]. Furthermore, this mechanism may be also responsible for the observed improvement in endothelial function in patients with T2DM following pharmacological inhibition of DPP4 with other gliptins (vildagliptin) [54]. All these effects mediated by DPP4 inhibition may confer some properties to gliptins that are related to reduction of endothelial damage and also to improvement in endothelial function, with possibly atheroprotective action.

## 5. DPP4 and Inflammation

DPP4 also seems to play an important role in low-grade inflammation [55] and particularly in the development of inflammatory reactions in patients with T2DM [15]. IR* per se* and the chronic low-grade inflammation present in T2DM may increase the expression and release of DPP4 from several tissues [15]. Indirect markers of IR, such as fasting insulin and homeostasis model assessment to quantify insulin resistance (HOMA-IR), were positively associated with DPP4 expression in visceral adipose tissue (VAT) macrophages [56]. These macrophages, as well as the visceral adipocytes, were able to release DPP4 when stimulated by TNF-*α* [56].

On the other hand, treatment of human vascular endothelial cells with sitagliptin is able to inhibit TNF-*α* induction of plasminogen activator inhibitor type-1 (PAI-1), intercellular adhesion molecule-1 (ICAM-1), and vascular cell adhesion molecule-1 (VCAM-1) mRNA and protein expression [17]. DPP4 inhibition is also able to decrease serum levels of inflammatory cytokines, such as interleukin-6 and interleukin-18, in patients with T2DM [18]. Taken together, these evidences suggest the existence of a pathophysiological interaction between DPP4, endothelial dysfunction, and inflammation, factors that are directly linked to the pathogenesis and clinical manifestations of T2DM and atherosclerosis.

## 6. DPP4 as an Adipokine

Adipose tissue (AT) is definitely an endocrine organ. It expresses and secretes several proteins, known as adipokines, as well as inflammatory cytokines [57, 58]. Adipokines and cytokines participate in the main pathophysiological mechanism linking obesity, IR, T2DM, and atherosclerotic disease [58, 59]. Recently, DPP4 was identified as a new adipokine, possibly linking AT to IR and the metabolic syndrome [21].

In a series of basic and clinical researches, including a proteomic profile of human adipocyte, it was demonstrated that (1) DPP4 is highly expressed in and released by adipocytes; (2) DPP4 inhibits insulin-stimulated Akt phosphorylation in muscle and adipocyte, therefore impeding insulin signaling, and this effect was totally reversed by a DPP4 inhibitor which strongly suggest its role in IR; (3) DPP4 levels are higher in obese as compared to lean subjects and its expression is increased in VAT of obese when compared to subcutaneous adipose tissue (SAT) of obese or lean subjects; (4) DPP4 concentration correlated with several biochemical parameters, such as insulin, leptin (directly), and adiponectin (inversely) [21]. To further refine these observations, Sell et al. [60] studied DPP4 expression and release by VAT and SAT in a cohort of 196 subjects before an open abdominal surgery, by collecting AT biopsies. These authors demonstrated a positive relationship between DPP4 expression and body mass index in both SAT and VAT, with VAT exhibiting higher expression. Furthermore, VAT released more DPP4 than SAT. Interestingly, DPP4 serum levels were higher in insulin resistant as compared to insulin sensitive subjects matched for BMI. Taken together, these data demonstrated that DPP4 is a new adipokine associated with increased visceral obesity, IR, and metabolic syndrome, which are all well-known risk factors for atherosclerotic disease.

**Figure 1:**
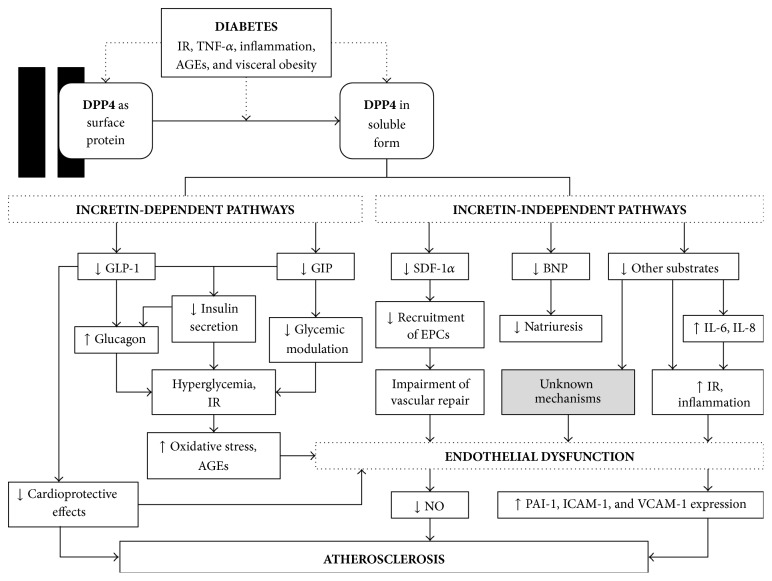
Schematic diagram illustrating the role of DPP4 and its associations with diabetes, insulin resistance, and atherosclerosis. AGEs: advanced glycation end products; BNP: B-type natriuretic peptide; DPP4: dipeptidyl peptidase 4; EPCs: endothelial progenitor cells; GIP: glucose-dependent insulinotropic polypeptide; GLP-1: glucagon-like peptide 1; ICAM-1: intercellular adhesion molecule-1; IL-6: interleukin-6; IL-8: interleukin-8; IR: insulin resistance; NO: nitric oxide; PAI-1: plasminogen activator inhibitor type-1; SDF-1*α*: stromal cell-derived factor 1*α*; TNF-*α*: tumor necrosis factor *α*; VCAM-1: vascular cell adhesion molecule-1.

Figure 1 provides a schematic diagram illustrating the above-mentioned associations between DPP4, T2DM, insulin resistance, and atherosclerosis.

## 7. Impact of DPP4 Inhibition on Atherosclerotic Cardiovascular Disease: Some Clinical Aspects

Several DPP4 inhibitors have been launched in the market and are now being used for the treatment of T2DM (vildagliptin, sitagliptin, saxagliptin, linagliptin, and alogliptin) [7]. All of them proved efficacy in glycemic control with impressive safety and tolerance profiles [26]. Gliptins can be used as monotherapy or in combination with other oral agents (in dual or triple therapy) and even with insulin [61]. A systematic review and meta-analyses showed similar efficacy and safety for gliptins as monotherapy or as combination therapy for T2DM [62].

As add-on therapy to metformin, DPP4 inhibitors reduced mean A1c by 0.5–1.1% compared with placebo [63–66]. Considering the glycemic control achieved by a combination of metformin with sulfonylureas or glitazones, gliptins provided comparable improvements [67–69], although with reduced risk of hypoglycemia and weight gain when compared to sulphonylureas and greater cost to the patients. However, in a recent retrospective analysis, gliptins in combination with metformin showed better metabolic control, lower rates of hypoglycemia, and even lower health costs in comparison to metformin and other oral agents in subjects with T2DM and renal impairment [70]. A study [71] comparing gliptins, sulphonylureas, insulin, and GLP-1 receptor agonists for use after metformin is ongoing and will possibly add knowledge about the most appropriate drugs for the treatment of T2DM.

Several meta-analyses of phase III randomized clinical trials (RCTs) have been published evaluating the impact of DPP4 inhibitors on CV outcomes. Frederich et al. [72] and Johansen et al. [73] showed, respectively, that saxagliptin and linagliptin were able to reduce CV outcomes (hazard ratio (HR) from 0.34 to 0.43) when compared to other agents, including placebo. No differences in CV events were observed by Schweizer et al. [74] and Engel et al. [75] when, respectively, vildagliptin and sitagliptin were compared to other oral drugs or placebo. Eighteen RCTs were analyzed together in a meta-analysis that included 8544 patients treated for at least 24 weeks with gliptins or other oral antidiabetic drugs. These investigators found that gliptins may possibly reduce risk of adverse CV events by observing a RR of 0.48 (95% CI: 0.31–0.75; *P* = 0.001) for any adverse CV event and a RR of 0.40 (95% CI: 0.18–0.88, *P* = 0.02) for nonfatal myocardial infarction (MI) or acute coronary syndrome in those treated with a DPP4 inhibitor [76]. Similar results were obtained by Monami et al. [61] in a meta-analysis enrolling almost 42000 patients with T2DM. They found that gliptins promoted a 29% reduction in major cardiovascular events (MACE), mostly due to reducing MI (<36%) and all-cause mortality (<40%). Individually, vildagliptin and saxagliptin were associated with less MACE [61].

Questions about the validity of these comparisons must be taken in account, since many pitfalls in primary composite endpoints and CV adjudication methods were noted [73]. It is also not clear how these potential benefits may be mediated, but possibly these drugs acted through the improvement in endothelial function, inflammation, and reduction of atherosclerosis [77]. Interestingly, an* in vivo* experiment recently demonstrated that vildagliptin or sitagliptin reduced MI size in rats in a glucose-dependent manner through GLP-1 receptor-protein kinase A pathway [78], while linagliptin attenuated neointima formation after vascular injury and* in vitro* vascular smooth muscle cells proliferation beyond the glucose-lowering effect [79].

Table 2 shows some characteristics of phase IV clinical trials evaluating the impact of long-term DPP4 inhibition on CV outcomes. In RCTs designed to demonstrate noninferiority, alogliptin and saxagliptin were neutral regarding MACE [22, 23]. In the EXAMINE trial [22], involving patients with T2DM who had recent hospitalization for acute coronary syndrome, MACE rates did not differ between those who used alogliptin compared to placebo (HR: 0.96; upper boundary of the one sided repeated confidence interval: 1.16; *P* = 0.32 for superiority; *P* < 0.001 for noninferiority) after a follow-up period greater than 40 months (median of 18 months). In the SAVOR-TIMI 53 trial [23], DPP4 inhibition with saxagliptin did not alter the rate of CV events (HR: 1.0; 95% CI: 0.89 to 1.12; *P* = 0.99 for superiority; *P* < 0.001 for noninferiority), although a higher heart failure hospitalization rate among saxagliptin users has been detected (HR: 1.27; 95% CI: 1.07–1.51; *P* = 0.007) during follow-up (median 2.1 years).

The increased risk of hospitalization for heart failure associated with the use of gliptins still requires further analysis. In a later study with SAVOR-TIMI 53 data [80], it was demonstrated that although the absolute risk of hospitalization for heart failure was highest among the 12.8% of patients who had a history of this condition, the relative risk of hospitalization for the same cause among patients assigned to saxagliptin was similar regardless of the baseline history (HR: 1.21; 95% CI: 0.93–1.58 versus HR: 1.32; 95% CI: 1.04–1.65; *P* = 0.68 for interaction). Moreover, in a reanalysis of the EXAMINE trial [81], including patients with a history of heart failure and/or high baseline levels of N-terminal pro-B-type natriuretic peptide, there was no evidence of an increased risk of CV outcomes or the rate of hospitalization for heart failure among patients assigned to alogliptin compared to placebo. During follow-up, alogliptin did not induce the onset of heart failure in patients without this diagnosis, or worsening of symptoms in patients with this previous diagnosis [81].

Despite these evidences, in a recent meta-analysis of 94 RCTs enrolling 85224 patients, including data from SAVOR-TIMI 53 and EXAMINE trials, Savarese et al. [82] observed that gliptins did not affect all-cause and CV mortality, as well as stroke, both in short- (<29 weeks) and long-term (≥29 weeks) therapies. With respect to the risk of MI, they also noted that gliptins reduced this risk in short-term treatment (RR: 0.58; 95% CI: 0.36–0.94; *P* = 0.02), but it did not persist in the long-term. Furthermore, long-term treatment with gliptins was associated with a 15.8% increase in the risk of heart failure (RR: 1.15; CI: 1.01–1.32; *P* = 0.03). So, it is still not possible to rule out the existence of an interaction between DPP4 inhibition and heart failure. As mentioned above, B-type natriuretic peptide (BNP) and substance P are both substrates of the DPP4 enzyme and may have implications on the possible association between heart failure and gliptins use, since it is already known that BNP levels increased more than 100 times in patients with heart failure and substance P is able to increase sympathetic activity during combined inhibition of angiotensin-converting enzyme and DPP4 [30, 77, 83, 84].

Regarding the risk of MI, patients enrolled in RCTs assessing the CV safety of gliptins have some characteristics that could be responsible for the observed diversities in the obtained results. To investigate it, Dicembrini and Mannucci [85] performed a meta-analysis with RCTs designed for glycemic endpoints that had a duration of 52 weeks or longer. All RCTs were identified from Savarese et al. [82], except those studies with a CV endpoint. During a mean follow-up of 86.3 weeks, gliptins were associated with a significant reduction of MI (RR: 0.48; 95% CI: 0.31–0.73; *P* = 0.001) similar to that observed in short-term therapy by Savarese et al. [82]. The authors concluded that maybe gliptins have a protective effect only in earlier stages of the natural history of T2DM (i.e., in younger subjects with a short duration of disease and without an established CV disease) whereas this benefit is lost in older patients with already established CV disease [85]. Therefore, there seems to be a window of opportunity for gliptins to reduce CV outcomes in subjects with T2DM that must be further investigated with studies primarily aimed at CV outcomes. The CAROLINA [24] and TECOS [25] trials, involving, respectively, linagliptin and sitagliptin, are still in progress and will possibly provide additional important information about the impact of pharmacological inhibition of DPP4 on CV outcomes.

## 8. Conclusion

The activity of DPP4 seems to be increased in patients with T2DM and there are a fair number of* in vitro* and* in vivo* studies demonstrating that this enzyme is able to interact with proinflammatory pathways and to impair endothelial function through incretin-dependent and independent mechanisms, potentially providing a new link between T2DM and atherosclerosis. In this way, it has been demonstrated that DPP4 is a new adipokine associated with increased visceral obesity, IR, and metabolic syndrome, which is consistent with its possible link with atherosclerosis. Many studies showed that DPP4 inhibition attenuated endothelial dysfunction, inflammation, and atherosclerotic process, but available phase IV studies did not associate the use of gliptins with reduced CV events in T2DM. In light of current evidence, we believe that further clinical studies with gliptins are warranted, especially those primarily aimed to investigate cardiovascular outcome.

## Figures and Tables

**Table 1:** Dipeptidyl peptidase 4 (DPP4) substrates.

*β*-Casomorphin-2	GLP-1^*^ and -2^*^	MCP
Aprotinin	Glucagon	MDC
Bradykinin	GRF	Morphiceptin
BNP^*^	GRP	Neuropeptide Y
CLIP	IGF-1	PACAP27
Chromogranin	IL-1*β*	PACAP38
Endomorphin-1	IL-2	Procalcitonin
Endomorphin-2	GCP-2 (CXCL6)	Peptide YY
Enterostatin	Mig (CXCL9)	PHM
Eotaxin (CCL11)	IP-10 (CXCL10)	RANTES (CCL5)
Monomeric fibrin (*α*-chain)	I-TAC (CXCL11)	Substance P^*^
GHRH	SDF-1*α* and -1*β* (CXCL12)^*^	Vasostatin I
GIP^*^	LD78*β* (CCL3L1)	VIP

BNP: B-type natriuretic peptide, formerly named brain natriuretic peptide; CLIP: corticotropin-like intermediate lobe peptide; GHRH: growth hormone-releasing hormone; GIP: glucose-dependent insulinotropic polypeptide; GLP-1: glucagon-like peptide 1; GLP-2: glucagon-like peptide 2; GRF: growth hormone-releasing factor; GRP: gastrin-releasing peptide; IGF-1: insulin-like growth factor 1; IL-1*β*: interleukin-1*β*; IL-2: interleukin-2; GCP-2: granulocyte chemotactic protein 2; IP-10: interferon *γ*-inducible protein 10; I-TAC: interferon *γ*-inducible T cell alpha chemoattractant; SDF-1*α*: stromal cell-derived factor 1*α*; SDF-1*β*: stromal cell-derived factor 1*β*; LD78*β*: isoform of macrophage inflammatory protein-1*α* (MIP-1*α*); MCP: monocyte chemotactic protein; MDC: macrophage-derived chemokine; PACAP27: pituitary adenylate cyclase-activating peptide 27; PACAP38: pituitary adenylate cyclase-activating peptide 38; PHM: peptide histidine methionine; RANTES: regulated on activation, normal T-cell expressed and secreted; VIP: vasoactive intestinal peptide. ^*^Peptides whose endogenous levels of intact to cleaved forms are significantly different following genetic inactivation or chemical inhibition of DPP4 activity *in vivo*. Adapted from [20, 28, 30].

**Table 2:** Prospective, randomized, controlled trials involving DPP4 inhibitors (gliptins) and cardiovascular outcomes in diabetic patients.

Gliptin versus comparator	Study	Doses (mg/day)	Composite primary endpoints	Population
Alogliptin versus placebo [22]	*Examination of cardiovascular outcomes with alogliptin versus standard of care* (*EXAMINE*)^*^	6.25, 12.5, or 25	Nonfatal MI, nonfatal stroke, or CV death	Patients with T2DM recently hospitalized for an ACS (*n* = 5380)

Saxagliptin versus placebo [23]	*Saxagliptin assessment of vascular outcomes recorded in patients with diabetes mellitus – thrombolysis in myocardial infarction 53 trial* (*SAVOR-TIMI 53*)^**^	2.5 or 5	Nonfatal MI, nonfatal ischemic stroke, or CV death	High-risk CV patients with T2DM (*n* = 16492)

Linagliptin versus glimepiride [24]	*Cardiovascular outcome study of the DPP-4 inhibitor linagliptin* (*CAROLINA*)^**^	5	Nonfatal MI, nonfatal stroke, hospitalization for unstable angina, or CV death	High-risk CV patients with T2DM (*n* = ~6000)

Sitagliptin versus placebo [25]	*Trial to evaluate cardiovascular outcomes after treatment with sitagliptin* (*TECOS*)^***^	50 or 100	Nonfatal MI, nonfatal stroke, or hospitalization for unstable angina	Patients with T2DM and previous CV disease (*n* = ~14000)

ACS: acute coronary syndrome; CV: cardiovascular; MI: myocardial infarction; T2DM: type 2 diabetes mellitus. ^*^Superiority trial. ^**^Noninferiority and superiority trial. ^***^Noninferiority trial.  ^¶^Ongoing study. This is adapted from [26, 27].

## Abbreviations

AGEs: Advanced glycation end products
AT: Adipose tissue
BMI: Body mass index
BNP: B-type natriuretic peptide
CD26: Cluster of differentiation 26
CI: Confidence interval
CV: Cardiovascular
DPP4: Dipeptidyl peptidase 4
EPCs: Endothelial progenitor cells
GIP: Glucose-dependent insulinotropic polypeptide
GLP-1: Glucagon-like peptide 1
HR: Hazard ratio
HOMA-IR: Homeostasis model assessment to quantify insulin resistance
ICAM-1: Intercellular adhesion molecule-1
IR: Insulin resistance
MACE: Major cardiovascular events
MI: Myocardial infarction
NO: Nitric oxide
PAI-1: Plasminogen activator inhibitor type-1
RAGE: Receptor for advanced glycation end products
RCTs: Randomized clinical trials
RR: Relative risk
SAT: Subcutaneous adipose tissue
SDF-1*α*: Stromal cell-derived factor 1*α*
T2DM: Type 2 diabetes
TNF-*α*: Tumor necrosis factor *α*
VAT: Visceral adipose tissue
VCAM-1: Vascular cell adhesion molecule-1.
